# Evaluation of Dietary Guideline Adherence and Risk of Sarcopenia in Elder Taiwanese

**DOI:** 10.1002/fsn3.70343

**Published:** 2025-05-26

**Authors:** Yih‐Jin Liou, Edward Giovannucci, Wu‐Chien Chien, Li‐Wei Wu

**Affiliations:** ^1^ Department of General Medicine Tri‐Service General Hospital, National Defense Medical Center Taipei Taiwan, ROC; ^2^ Department of Nutrition Harvard T.H. Chan School of Public Health Boston Massachusetts USA; ^3^ Department of Epidemiology Harvard T.H. Chan School of Public Health Boston Massachusetts USA; ^4^ Department of Medical Research Tri‐Service General Hospital, National Defense Medical Center Taipei Taiwan, ROC; ^5^ School of Public Health National Defense Medical Center Taipei Taiwan, ROC; ^6^ Division of Family Medicine, Department of Family and Community Medicine Tri‐Service General Hospital, National Defense Medical Center Taipei Taiwan, ROC; ^7^ Division of Geriatric Medicine, Department of Family and Community Medicine Tri‐Service General Hospital, National Defense Medical Center Taipei Taiwan, ROC

**Keywords:** dietary adequacy, dietary quality, healthy eating index, muscle mass, sarcopenia

## Abstract

Dietary strategies play an important role in determining longevity and healthy aging. There is an increasing awareness of dietary factors and sarcopenia, which presents as a decline in muscle mass and function, mainly in the senior population. The aim of this study is to explore the association of adherence to the Dietary Guideline (DG) on muscle health in the elder Taiwanese. We included 410 adults aged 65 or older, of whom 189 (46.1%) were women, in the Nutrition and Health Survey in Taiwan (NAHSIT) during 2014–2016 with comprehensive records of food frequency questionnaires and 24‐h dietary recalls, as well as measurements of dual‐energy X‐ray absorptiometry (DXA) for muscle mass and 8‐m gait speed for physical performance. A novel indicator, named Healthy Eating Index for Taiwanese (HEI‐TW), was developed as an amended version of HEI‐2015 to assess how dietary patterns adhere to the DG of Taiwan. Participants who fulfilled “low muscle mass” and “poor physical performance” defined by the Asian Working Group for Sarcopenia (AWGS) criteria were recognized as having sarcopenia; others, in contrast, were recognized as healthy controls. Seventy‐one (17.3%) participants categorized into the sarcopenia group had significantly lower mean scores of overall HEI‐TW, total vegetables, unrefined grains, and dairy products. The adjusted odds ratios (95% confidence intervals) for sarcopenia were 0.98 (0.95–1.00) for overall HEI‐TW, 0.78 (0.66–0.94) for total vegetables, and 0.85 (0.76–0.95) for dairy, respectively, for one unit increase in these components. In subgroup analysis stratified by sex and age, the overall HEI‐TW was inversely associated with the odds of sarcopenia among men and individuals aged 75 or older. This study showed that a higher HEI‐TW score adhering to Taiwan DG and abundant consumption of total vegetables and dairy products is associated with lower prevalence of muscle loss and sarcopenia in elder Taiwanese.

## Graphical abstract

We utilized a nationwide nutrition survey in Taiwan and developed a novel index to explore the association between dietary adherence and sarcopenia. Better adherence to the dietary guideline, especially meeting the recommendations of vegetable and dairy intake, has an association with a lower risk of sarcopenia. By adapting a dietary index, this study provides a modified tool for assessing diet quality and its impact on sarcopenia in Taiwanese older adults.

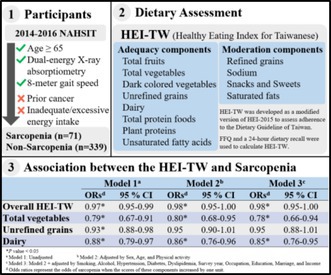

## 1 Introduction

With growing numbers of older people, increases in prevalence in geriatric illnesses and sarcopenia are inevitable (Beaudart et al. 2014). Sarcopenia is primarily characterized by a loss of muscle mass and by a functional decline, along with complications such as frailty, accidental falls, fractures, and mortality (Woo et al. 2015). Global prevalence estimates in adults range from 10% to 27% using various definitions and are presumably increasing over time with aging populations (Petermann‐Rocha et al. 2022; Ethgen et al. 2017). Studies have shown that the underlying mechanisms of decreased appetite and deteriorating digestive functions, which are common problems most senior individuals experience, lead to inadequate energy intake of nutrients and, as a result, have a negative effect on muscle mass and function (Tieland et al. 2018). To prevent sarcopenia and promote quality of life in the elderly, it is essential to sustain an optimal and well‐balanced nutritional status.

Among various methods to assess nutritional status, dietary patterns, which refer to the quantities, proportions, variety, or combination of different foods, beverages, and nutrients in diets, provide a comprehensive understanding of one's dietary habit. The Healthy Eating Index (HEI), an indicator used to quantify the adherence of people's dietary patterns to DG for Americans, was initially developed in 2005 and continuously updated by the U.S. Department of Agriculture (USDA) (Krebs‐Smith et al. 2018). Due to variations in dietary recommendations across different ethnicities and countries, previous studies have developed amended versions of the index, such as Alternative‐HEI (McCullough et al. 2002), and found associations with health status and several diseases (Onvani et al. 2017). However, there is limited relevant research on sarcopenia. The first study investigating the association between HEI and sarcopenia observed an inverse association in women but not in men. These analyses were only based on a small population in a particular area, and the results might therefore not be generalizable to other ethnicities or countries (Ghoreishy et al. 2023). Thus, whether HEI or other modified indexes are related to the risk of sarcopenia is still not clearly verified.

In this study, we aimed to evaluate the relationship between adherence to the Dietary Guideline (DG) of Taiwan and sarcopenia in the elderly. We developed a novel index, named Healthy Eating Index for Taiwanese (HEI‐TW), which is calculated similarly to the original HEI‐2015 but with modifications based on the DG of Taiwan (Krebs‐Smith et al. 2018; Health Promotion Administration, M.o.H.a.W 2016). A higher HEI‐TW score indicates better adherence to the DG and is hypothesized to have an association with a lower risk of sarcopenia.

## 2 Methods

### 2.1 Data Source and Participants

This is a cross‐sectional study using data from the Nutrition and Health Survey in Taiwan (NAHSIT) between 2014 and 2016. NAHSIT, launched by the Taiwan Ministry of Health and Welfare, is a nationwide nutrition survey monitoring the nutrition and health status of citizens (Health Promotion Administration, M.o.H.a.W 2024). To ensure an even distribution of gender, age, and residential area among study participants, a three‐stage probability sampling design was conducted, whose details have been described in previous publications (Chuang et al. 2019). Each participant had a face‐to‐face interview. Apart from basic sociodemographic data, a food frequency questionnaire and a 24‐h dietary recall were used to obtain dietary information. Participants aged 65 or older and completing assessments to determine sarcopenia, including measurement of 8‐m walk speed and dual energy X‐ray absorptiometry, were included. Exclusion criteria were the presence of a prior malignant cancer diagnosis, inadequate/excessive energy intake (defined as consuming less than 600 kcal or more than 4000 kcal a day), or missing information on HEI‐TW components. The Tri‐Service General Hospital Institutional Review Board granted the approval of a research study (TSGHIRB No. E202416017).

### 2.2 Dietary Assessment

In terms of dietary quality assessment, the HEI‐TW was designed as a 100‐point scale that encompasses two parts: (1) the adequacy components, including total fruits, total vegetables, dark colored vegetables, unrefined grains, dairy, unsaturated fatty acids, total protein foods, and plant proteins; and (2) the moderation components, including saturated fats, sodium, refined grains, and snacks and sweets. Daily food intake was primarily extracted from the 24‐h dietary recall, which documented the consumption of foods and nutrients by participants in the past 1 day. For the difficulty in differentiating between unrefined and refined grains in the 24‐h dietary recall, the food frequency questionnaire was applied to estimate the proportion of daily servings for refined and unrefined grains. For example, six servings of grains in the 24‐h dietary recall with three times of unrefined grains and six times of refined grains per week in the food frequency questionnaire were calculated as having two servings of unrefined grains and four servings of refined grains a day. The serving sizes of adequacy components, except for unsaturated fatty acids, for the maximum score were equivalent to or more than the recommended daily serving sizes from the DG of Taiwan, with a minimum score of zero for no serving (Health Promotion Administration, M.o.H.a.W 2016). The recommended daily serving sizes of components were dependent on the recommended daily calorie intake, which, in turn, was determined by sex, age, and level of physical activity. For example, a 55‐year‐old woman with moderate physical activity should aim to consume 1800 cal a day, which requires two servings of fruit to meet the recommended serving sizes and obtain the maximum score in the fruit component. The standards for the maximum and minimum score of unsaturated fatty acids, sodium, and saturated fats were in line with those on HEI‐2015 (Krebs‐Smith et al. 2018). With respect to refined grains, the ratio of serving sizes for the maximum and minimum score on HEI‐TW to those on HEI‐2015 is commensurate with the ratio of recommended daily serving sizes from the DG of Taiwan to those from DG for Americans. Moreover, due to the lack of information on the calories of added sugars, we replaced it with the calories of snacks and sweets, with the same standards of the maximum and minimum score for added sugars on HEI‐2015. The scores of all these components were summed up to calculate the total score of HEI‐TW. Details about the scoring and recommended daily calorie intake from the DG of Taiwan are presented in Tables S1 and S2.

### 2.3 Sarcopenia Assessment

The diagnosis of sarcopenia in the current study was adapted based on criteria from the AWGS 2019 consensus (Chen et al. 2020). Calculated by dual‐energy x‐ray absorptiometry, appendicular skeletal muscle mass lower than 7.0 kg/m^2^ in males and 5.7 kg/m^2^ in females was identified as low muscle mass. Gait speed measured on an 8‐m walk lower than 1 m/s was recognized as poor physical performance. Participants were divided into two groups: those with low muscle mass and poor physical performance were classified in the sarcopenia group, and others were in the healthy control group.

### 2.4 Statistical Analysis

The percentages, or mean and standard deviations, of basic characteristics and the HEI‐TW score in the healthy control and sarcopenia groups were examined, and their differences were contrasted by a chi‐square or independent *T*‐test. The associations between the presence of sarcopenia and the HEI‐TW were assessed through logistic regression analysis. Potential confounders, including age, gender, physical activity, habit of smoking and drinking, underlying disease, survey year, occupation, education, marriage status, and financial condition, were considered for further adjusted models. In subgroup analyses, participants were categorized in accordance with gender, age (75 years as a cutoff point for early elderly and late elderly), and BMI (24 kg/m^2^ as a cutoff point for non‐overweight and overweight). Statistical estimations were conducted using SPSS (SPSS Inc., Chicago, Illinois, USA), and statistical significance was indicated by a *p* value less than 0.05.

## 3 Results

Table 1 illustrates the demographic and clinical characteristics of all the participants. Among the 410 participants, 71 (17.3%) were categorized as having sarcopenia, and the rest, 339 (82.7%), were healthy controls, with the portion of male, late elderly, and non‐overweight being higher in the sarcopenia group. The distribution of other variables such as the habit of smoking and drinking, underlying diseases of hypertension, diabetes, and dyslipidemia, survey year, resident area, education level, marital status, and monthly income were similar between the two groups.

**TABLE 1:** Demographic and clinical characteristics of subjects.

	Healthy control (*n* = 339)	Sarcopenia (*n* = 71)	*p*
Sex			
Male	167	54	< 0.01
Female	172	17	
Age years (Mean/SD)	71.66/5.66	77.90/6.42	< 0.01
< 75	243	21	< 0.01
≥ 75	96	50	
Body mass index kg/m^2^ (Mean/SD)	25.09/3.56	22.44/2.63	< 0.01
< 24	138	52	< 0.01
≥ 24	201	19	
Survey year			
2014	101	22	0.43
2015	126	21	
2016	112	28	
Physical activity			
Sedentary	307	66	0.63
Light	1	0	
Moderate	24	5	
Vigorous	7	0	
Occupation			
Employed	103	13	0.04
Unemployed or retired	236	58	
Education			
Elementary or junior high school	217	51	0.28
Senior high school	83	16	
College	39	4	
Marriage			
Single or divorce	98	21	0.51
Marriage	241	50	
Income			
< 10,000	175	46	0.35
10,000–30,000	78	13	
30,000–50,000	30	4	
> 50,000	40	5	
Unknown	16	3	
Exposure			
Alcohol (Yes/No)	59/280	9/62	0.33
Cigarette (Yes/No)	93/246	27/44	0.07
Underlying disease			
Hypertension (Yes/No)	168/171	32/39	0.29
Dyslipidemia (Yes/No)	58/281	10/61	0.34
Diabetes (Yes/No)	65/274	14/57	0.51
Component of sarcopenia			
8 m speed (m/s) (Mean/SD)	0.97/0.25	0.76/0.18	< 0.01
Muscle mass (kg/m2) (Mean/SD)	6.90/1.06	6.13/0.72	< 0.01

*Note:* Statistical significance was indicated by a *p* value less than 0.05 and the values presented in red color font.

Comparisons of the HEI‐TW score between the two groups are listed in Table 2. The healthy control group had a higher mean score of overall HEI‐TW (56.57 vs. 51.55, *p* value = 0.005), signifying better adherence to the DG of Taiwan. Significant differences also existed in the mean scores of total vegetables (3.90 vs. 3.26, *p* value = 0.006), unrefined grains (5.50 vs. 3.85, *p* value = 0.006), and dairy scores (2.13 vs. 1.04, *p* value = 0.002). Table 3 demonstrates several associations between the HEI‐TW and the odds of sarcopenia. In the unadjusted models, the scores of overall HEI‐TW, total vegetables, unrefined grains, and dairy were inversely associated with sarcopenia. In the adjusted model, the risk of sarcopenia decreased by 3% for overall HEI‐TW (OR = 0.97, 95% CI = 0.95–0.99), by 25% for total vegetables (OR = 0.75, 95% CI = 0.61–0.92), and by 13% for dairy (OR = 0.87, 95% CI = 0.77–0.97), when the scores of these components increased by one unit. Next, subgroup regression analysis according to gender, age, and BMI was examined in both adjusted and unadjusted models. As shown in Table 4, there are significant associations between the scores of overall HEI‐TW and sarcopenia present in male, late elderly, and non‐overweight participants. The complete results of subgroup analysis between components of HEI‐TW and sarcopenia are provided in Table S3.

**TABLE 2:** Comparisons of the HEI‐TW between healthy control and sarcopenia.

	Healthy control (*n* = 339)		Sarcopenia (*n* = 71)		*p*
	Mean	SD	Mean	SD	
Overall	56.57	13.69	51.55	13.41	< 0.01
Adequacy component					
Total fruits	5.92	3.90	4.94	4.06	0.06
Total vegetables	3.90	1.49	3.26	1.81	< 0.01
Dark colored vegetables	2.93	2.01	2.49	1.94	0.09
Unrefined grains	5.50	4.67	3.85	4.47	< 0.01
Dairy	2.13	3.35	1.04	2.55	< 0.01
Total protein foods	4.25	1.34	3.93	1.52	0.10
Plant proteins	1.78	2.26	1.94	2.19	0.59
Unsaturated fats	7.77	2.79	7.53	3.17	0.51
Moderation component					
Refined grains	1.37	3.25	1.39	3.23	0.97
Sodium	4.72	4.18	4.88	4.28	0.77
Snack and sweets	7.78	3.37	7.63	3.62	0.74
Saturated fats	8.52	2.50	8.67	2.50	0.63

*Note:* Statistical significance was indicated by a *p* value less than 0.05 and the values presented in red color font.

**TABLE 3:** Association between the HEI‐TW and sarcopenia.

	Model 1^a^			Model 2^b^			Model 3^c^		
	*p*	ORs^d^	95% CI	*p*	ORs^d^	95% CI	*p*	ORs^d^	95% CI
Overall	< 0.01	0.97	0.95–0.99	0.04	0.98	0.95–1.00	0.04	0.98	0.95–1.00
Adequacy component									
Total fruits	0.06	0.94	0.88–1.00	0.15	0.95	0.88–1.02	0.20	0.95	0.88–1.03
Total vegetables	< 0.01	0.79	0.67–0.91	0.01	0.80	0.68–0.95	< 0.01	0.78	0.66–0.94
Dark colored vegetables	0.09	0.90	0.79–1.02	0.13	0.90	0.78–1.03	0.15	0.90	0.77–1.04
Unrefined grains	< 0.01	0.93	0.88–0.98	0.13	0.95	0.90–1.01	0.10	0.95	0.88–1.01
Dairy	0.01	0.88	0.79–0.97	< 0.01	0.86	0.76–0.96	< 0.01	0.85	0.76–0.95
Total protein foods	0.07	0.86	0.72–1.01	0.21	0.88	0.73–1.07	0.25	0.89	0.73–1.09
Plant proteins	0.58	1.03	0.92–1.16	0.15	1.10	0.97–1.25	0.13	1.11	0.97–1.26
Unsaturated fats	0.51	0.97	0.89–1.06	0.70	0.98	0.89–1.08	0.78	0.99	0.89–1.09
Moderation component									
Refined grains	0.97	1.00	0.93–1.08	0.40	1.04	0.95–1.14	0.42	1.04	0.95–1.15
Sodium	0.77	1.01	0.95–1.07	0.91	1.00	0.94–1.07	0.74	1.01	0.94–1.08
Snack and sweets	0.74	0.99	0.92–1.06	0.59	0.98	0.90–1.06	0.50	0.97	0.89–1.06
Saturated fats	0.63	1.03	0.92–1.14	0.94	1.00	0.89–1.13	1.00	1.00	0.88–1.13

*Note:* Statistical significance was indicated by a *p* value less than 0.05 and the values presented in red color font.

Abbreviations: CI, confidence intervals; ORs, odds ratios.

^a^ Model 1: Unadjusted.

^b^ Model 2: Adjusted by sex, age, and physical activity.

^c^ Model 3: Model 2 + adjusted by smoking, alcohol, hypertension, diabetes, dyslipidemia, survey year, occupation, education, marriage, and income.

^d^ Odds ratios represent the odds of sarcopenia when the scores of these components increased by one unit.

**TABLE 4:** Subgroup analysis of association between the score of overall HEI‐TW and sarcopenia.

	Model 1^a^			Model 2^b^			Model 3^c^		
	*p*	ORs^d^	95% CI	*p*	ORs^d^	95% CI	*p*	ORs^d^	95% CI
Sex									
Male	0.02	0.97	0.95–1.00	0.05	0.97	0.94–1.00	0.03	0.97	0.94–1.00
Female	0.17	0.98	0.94–1.01	0.21	0.98	0.94–1.01	0.17	0.97	0.93–1.01
Age									
< 75	0.9	0.98	0.95–1.02	0.32	0.98	0.95–1.02	0.65	0.99	0.96–1.03
≥ 75	0.04	0.97	0.94–1.00	0.06	0.97	0.94–1.00	0.05	0.97	0.93–1.00
Body mass index									
< 24	< 0.01	0.96	0.93–0.98	0.03	0.97	0.94–1.00	0.12	0.98	0.95–1.01
≥ 24	0.18	0.98	0.94–1.01	0.17	0.97	0.93–1.01	0.06	0.96	0.92–1.00

*Note:* Statistical significance was indicated by a *p* value less than 0.05 and the values presented in red color font.

Abbreviations: CI, confidence intervals; ORs, odds ratios.

^a^ Model 1: Unadjusted.

^b^ Model 2: Adjusted by sex, age, and physical activity.

^c^ Model 3: Model 2 + adjusted by smoking, alcohol, hypertension, diabetes, dyslipidemia, survey year, occupation, education, marriage, and income.

^d^ Odds ratios represent the odds of sarcopenia when the scores of these components increased by one unit.

## 4 Discussion

Diet is hypothesized to influence the risk of sarcopenia in the elderly. The HEI, in accordance with DG for Americans, is effective in quantifying dietary patterns, but its applicability to Taiwanese may be limited due to differences in dietary patterns and customs. The DG of Taiwan provides daily dietary recommendations and is widely known by most Taiwanese. To develop a diet quality scoring tool tailored for the Taiwanese population, we modified the HEI‐2015 based on the principles of the DG of Taiwan into a novel index, named HEI‐TW. Scores of the overall HEI‐TW, total vegetables, and dairy were associated inversely with the odds of sarcopenia. The results, if shown to be causal, suggest that dietary patterns more adherent to the DG of Taiwan may reduce or ameliorate the occurrence of sarcopenia in older populations.

The relationships between dietary patterns and age‐related loss of muscle mass and strength have been widely investigated. For instance, the Mediterranean diet, which focuses on plant‐based foods and healthy fats, has been associated with the lower loss of muscle mass and function (Isanejad et al. 2018; Cervo et al. 2021). Few studies have shown that the Dietary Approaches to Stop Hypertension (DASH) diet and plant‐based diets decrease the risk of sarcopenia (Perry et al. 2022; Chan et al. 2021). People consuming more vegetables, fruits, high‐quality protein, whole grains, nuts, and seeds were demonstrated to have greater muscle mass (Sabir et al. 2023). In contrast, a diet abundant in red meat, butter, and sweets showed the opposite association (Granic, Mendonça, et al. 2020). Dietary factors associated with sarcopenia were demonstrated in eastern‐based research studies as well. The Japanese dietary pattern, characterized by soy products, fish, vegetables, miso soup, and green tea, was correlated with a low prevalence of muscle weakness in middle‐aged and older Japanese individuals (Shimizu et al. 2022, 2023). In a nationwide cross‐sectional study undertaking a 24‐h dietary recall among Koreans, an inverse association was observed between the dietary pattern based on white rice, fish, and seaweed and low muscle mass in both the male and female populations (Kim et al. 2020). In residents of China, the prevalence of sarcopenia was negatively related to plant‐based, vegetarian, and dairy diet patterns (Chen et al. 2021).

The governments of most countries release food guidelines to educate their citizens on how to eat a healthy diet. Those recommendations are intended to advance the prevention of certain diseases, while more evidence is required to verify whether following a food guide brings advantages for muscle health. To measure the alignment of people's diet with the DG of Taiwan, we referred to and modified the HEI‐2015 and found that participants with better adherence to the recommended daily intakes had a lower risk of sarcopenia. Similar results in different populations were shown in studies with the HEI or amended versions of the index. Two separate studies in Iran utilized food‐frequency questionnaires to examine the quality of diet among elderly women, one of which found a positive connection between HEI‐2015 and muscle mass (Kamari et al. 2023), and the other found an inverse connection between Alternative HEI‐2010 and the odds of sarcopenia (Ghoreishy et al. 2023). The Korean Healthy Eating Index (KHEI), which has almost the same components as HEI‐2015, was developed to assess the overall dietary quality of Korean adults (Yun et al. 2022). Significant associations between KHEI scores and sarcopenia were reported based on data from the national nutrition survey in Korea (Lee and Park 2023).

Chronic inflammation, a condition often accompanied by metabolic syndrome, may be a responsible factor involving the association between nutrition and sarcopenia (Sayer et al. 2013). People with sarcopenia have a higher level of circulating inflammatory markers, such as CRP, IL‐6, IL‐10, and TNF‐α (Shokri‐Mashhadi et al. 2021; Rong et al. 2018; Chang et al. 2023). Increased free radicals can interfere with the balance between the anabolism and catabolism of proteins, which brings about muscle cell aging and damage (Meng and Yu 2010). Consuming adequate antioxidants from the diet such as flavonoids, carotenoids, vitamin C, and vitamin E may alleviate the effects of inflammation (Damiano et al. 2019). Fruits and vegetables are rich in those nutrients and are correlated with a lower level of inflammatory markers (Koyanagi et al. 2020), and possibly may ameliorate inflammation‐induced muscle loss and reduce the prevalence of sarcopenia. Several studies in East Asia showed that fruit and vegetable intake is associated with a lower risk of sarcopenia (Wang et al. 2022). In our study, the odds of sarcopenia were significantly related to the score of vegetables and borderline significantly related to the score of fruits, which were in line with the findings of previous studies.

Another component of HEI‐TW relating to the risk of sarcopenia in our study is dairy, generally considered a nutrient‐rich food with essential supplements of protein, calcium, phosphorus, and vitamin D (Du et al. 2019). Protein, calcium, and phosphorus prompt muscle mass and strength gains through various pathways. Vitamin D assists in the absorption of calcium and phosphorus in the intestine, as well as calcium transport and protein synthesis in muscle cells (Pfeifer et al. 2002). An increase in serum insulin‐like growth factor‐1 and growth hormone levels, considered to protect against sarcopenia (Bian et al. 2020), is also associated with a higher intake of dairy (Giovannucci et al. 2003). Several interventional and observational studies have reported that sufficient intake or supplement of those nutrients can potentially enhance muscle mass and function. Bioactive lipids, fatty acids, and peptides derived from dairy may reduce the inflammation in muscles (Granic, Hurst, et al. 2020). A meta‐analysis of eight randomized controlled trials found a modest improvement in appendicular muscle mass in the group receiving more dairy supplement (Hanach et al. 2019). A prospective observational studies conducted, respectively, in Korea showed an inverse association between dairy protein intake and low muscle mass in middle‐aged men (So and Joung 2020). A Spanish prospective study reported a lower risk of slow walking speed in elders taking seven or more servings of dairy products a week (Lana et al. 2015). In an intervention trial, participants with more cheese consumption had higher skeletal muscle mass than those without (Alemán‐Mateo et al. 2014). Consistent results were also seen in a cross‐sectional study of Taiwanese elderly (Kuo et al. 2022). The abundance of other vitamins and minerals in dairy also helps to fill dietary nutrient gaps (Górska‐Warsewicz et al. 2019), especially among the elderly, who often experience malnutrition due to poor appetite and declining abilities to chew and swallow. In the last NAHSIT data set, 64.3%–92.6% of men and 91.4%–93.8% of women aged 65 or older consumed less than one serving of dairy per day, which indicated a large portion of Taiwanese did not achieve the recommended 1.5 servings of daily dairy consumption (Health Promotion Administration, M.o.H.a.W 2022).

Male, older, and lower BMI participants are prone to developing sarcopenia, which is consistent with our results. Studies on Asian populations presented discrepant results on the prevalence of sarcopenia in the elderly, while most studies observed higher incidences in advancing age and men (Hwang and Park 2022; Chen et al. 2014; Lau et al. 2005). Additionally, without alterations in body composition, people with a higher BMI have both more fat and muscle mass. Because aging is usually accompanied by a lower metabolism rate and more fat accumulation, BMI is not an optimal classification for adiposity for older people; nonetheless, the elderly with underweight or normal weight seem to present a higher likelihood of sarcopenia (Lau et al. 2005; Liu et al. 2023). Results in subgroup analysis also showed significant associations between HEI‐TW and sarcopenia in male, late elderly, and non‐overweight populations, suggesting better adherence to DG is more essential for those with demographically high‐risk factors.

Some limitations must be acknowledged in this study. Due to the cross‐sectional observational research design, a cause‐and‐effect relationship between HEI‐TW and sarcopenia is not able to be established. Next, the participants included in the analysis were in relatively small numbers. Extended investigations are warranted to verify the results and generalize them to a broader population. Further, dietary data were collected based on the participants' recall of diet in the last 24 h and the food frequency questionnaire, whose validity and authenticity may be affected by various factors, especially in seniors with declines in cognitive function. In addition, our research indicated that adhering to DG, which focuses on chronic diseases generally, reduces the risk of sarcopenia; however, this does not necessarily imply that it constitutes the most optimized dietary pattern for preventing sarcopenia. Foods not specifically recommended in DGs, such as animal protein sources, can also improve muscle mass and might contribute to muscle health. Last, the DG of Taiwan did not provide precise recommendations for some components of HEI‐2015, such as fatty acids, sodium, added sugars, and saturated fats, which makes it challenging to set cutoff points. We used the same standards for scoring HEI‐2015 in those components and replaced added sugars with snacks and sweets. Thus, the definition of HEI‐TW is not completely based on the DG of Taiwan, while it can serve as an indicator of healthy eating.

## 5 Conclusions

HEI‐TW is a novel index to evaluate the influence of adherence to DG of Taiwan on the development of sarcopenia in the Taiwanese elderly. Dietary strategies for longevity and healthy aging, especially meeting the recommendations of vegetable and dairy intake, may have beneficial effects on the prevention of muscle loss and sarcopenia in population aging.

## Author Contributions

**Yih‐Jin Liou:** writing – original draft (equal). **Edward Giovannucci:** writing – review and editing (equal). **Wu‐Chien Chien:** data curation (equal), formal analysis (equal). **Li‐Wei Wu:** conceptualization (equal), writing – original draft (equal), writing – review and editing (equal).

## Ethics Statement

The study protocol was approved by the Tri‐Service General Hospital Institutional Review Board, which granted the approval of a research study (TSGHIRB No. E202416017).

## Conflicts of Interest

The authors declare no conflicts of interest.

## Supporting information


Appendix S1.

## Data Availability

The datasets generated and analyzed during the current study are not publicly available due to the institution's policy but will be available through contacts with the corresponding author on reasonable requests.
