# Supplementary material for: Evaluation of Dietary Guideline Adherence and Risk of Sarcopenia in Elder Taiwanese
Source: Food Sci Nutr. 2025 May 26;13(6):e70343. doi: 10.1002/fsn3.70343 (PMC12121529; doi:10.1002/fsn3.70343)
Supplement: Supplementary file 1 — Appendix S1. [file FSN3-13-e70343-s001.docx]

**Supplementary table 1**

| **Healthy Eating Index for Taiwanese (HEI-TW)** | | | | | | | | | | | | | | | | | |  |
| --- | --- | --- | --- | --- | --- | --- | --- | --- | --- | --- | --- | --- | --- | --- | --- | --- | --- | --- |
| **Component** | | **Maximum**  **points** |  | **Recommended daily calorie intake ^a^** | | | | | | | | | | | | | | |
|  |  |  |  | 1200 kcal | | 1500 kcal | | 1800 kcal | | 2000 kcal | | 2200 kcal | | 2500 kcal | | 2700 kcal | | |
|  |  |  |  | maximum score | score  of zero | maximum score | score  of zero | maximum score | score  of zero | maximum score | score  of zero | maximum score | score  of zero | maximum score | score  of zero | maximum score | score  of zero | |
| **Adequacy ^b^** | |  |  |  |  |  |  |  |  |  |  |  |  |  |  |  |  | |
| Total fruits (servings) | | 10 |  | 2 | 0 | 2 | 0 | 2 | 0 | 3 | 0 | 3.5 | 0 | 4 | 0 | 4 | 0 | |
| Total vegetables (servings) | | 5 |  | 3 | 0 | 3 | 0 | 3 | 0 | 4 | 0 | 4 | 0 | 5 | 0 | 5 | 0 | |
| Dark colored vegetables (servings) | | 5 |  | 1.5 | 0 | 1.5 | 0 | 1.5 | 0 | 2 | 0 | 2 | 0 | 2.5 | 0 | 2.5 | 0 | |
| Unrefined grains (servings) | | 10 |  | 1 | 0 | 1 | 0 | 1 | 0 | 1 | 0 | 1.5 | 0 | 1.5 | 0 | 1.5 | 0 | |
| Dairy (servings) | | 10 |  | 1.5 | 0 | 1.5 | 0 | 1.5 | 0 | 1.5 | 0 | 1.5 | 0 | 1.5 | 0 | 2 | 0 | |
| Total protein foods (servings) | | 5 |  | 3 | 0 | 4 | 0 | 5 | 0 | 6 | 0 | 6 | 0 | 7 | 0 | 8 | 0 | |
| Plant proteins (servings) | | 5 |  | 1 | 0 | 1.3 | 0 | 1.7 | 0 | 2 | 0 | 2 | 0 | 2.3 | 0 | 2.7 | 0 | |
| Unsaturated fats  ((PUFAs + MUFAs)/SFAs) | | 10 |  | 2.5 | 1.2 | 2.5 | 1.2 | 2.5 | 1.2 | 2.5 | 1.2 | 2.5 | 1.2 | 2.5 | 1.2 | 2.5 | 1.2 | |
| **Moderation ^c^** | |  |  |  |  |  |  |  |  |  |  |  |  |  |  |  |  | |
| Refined grains (servings) | | 10 |  | 0.6 | 1.4 | 1.8 | 4.2 | 2.4 | 5.6 | 2.4 | 5.6 | 2.4 | 5.6 | 3 | 7 | 3 | 7 | |
| Sodium (g/1,000 kcal) | | 10 |  | 1.1 | 2 | 1.1 | 2 | 1.1 | 2 | 1.1 | 2 | 1.1 | 2 | 1.1 | 2 | 1.1 | 2 | |
| Snack and sweets (% of energy) | | 10 |  | 6.5 | 26 | 6.5 | 26 | 6.5 | 26 | 6.5 | 26 | 6.5 | 26 | 6.5 | 26 | 6.5 | 26 | |
| Saturated fats (% of energy) | | 10 |  | 8 | 16 | 8 | 16 | 8 | 16 | 8 | 16 | 8 | 16 | 8 | 16 | 8 | 16 | |
| ^a.^ | The Recommended Daily Calorie Intake is determined by gender, age, and activity level (provided in Supplementary table 2). Each participant is assigned to the group in HEI-TW whose energy intake is equal to or is smaller but closest to their Recommended Daily Calorie Intake. For instance, a 65-year-old man with light physical activity is categorized in the 1800 kcal group. | | | | | | | | | | | | | | | | | |
| ^b.^ | In the scoring of adequacy components, servings or percentages higher than those for maximum score are assigned a maximum score, and no intake or percentages lower than those for score of zero receive zero points. Servings or percentages between those for maximum score and score of zero are scored in proportion. | | | | | | | | | | | | | | | | | |
| ^c.^ | In the scoring of moderation components, servings or percentages lower than those for maximum score are assigned a maximum score, and servings or percentages higher than those for score of zero receive zero points. Servings or percentages between those for maximum score and score of zero are scored in proportion. | | | | | | | | | | | | | | | | | |

**Supplementary table 2**

| **Recommended daily calorie intake ^a^** | | | | | | |
| --- | --- | --- | --- | --- | --- | --- |
|  | | Age | Physical activity | | | |
|  |  |  | Sedentary | Light | Moderate | Vigorous |
| Male | | 19-30 | 1850 kcal | 2150 kcal | 2400 kcal | 2700 kcal |
|  |  | 31-51 | 1800 kcal | 2100 kcal | 2400 kcal | 2650 kcal |
|  |  | 51-70 | 1700 kcal | 1950 kcal | 2250 kcal | 2500 kcal |
|  |  | ≥ 71 | 1650 kcal | 1900 kcal | 2150 kcal |  |
| Female | | 19-30 | 1500 kcal | 1700 kcal | 1950 kcal | 2150 kcal |
|  |  | 31-51 | 1450 kcal | 1650 kcal | 1900 kcal | 2100 kcal |
|  |  | 51-70 | 1400 kcal | 1600 kcal | 1800 kcal | 2000 kcal |
|  |  | ≥ 71 | 1300 kcal | 1500 kcal | 1700 kcal |  |
| ^a.^ | Based on Daily Food Guides of Taiwan: <https://www.hpa.gov.tw/Pages/EBook.aspx?nodeid=1208> | | | | | |

**Supplementary table 3**

**Subgroup analysis of association between the HEI-TW and Sarcopenia**

|  | **Male** | | | | | | | | | | | | | | | | | |  | **Female** | | | | | | | | | | | | | | | | |
| --- | --- | --- | --- | --- | --- | --- | --- | --- | --- | --- | --- | --- | --- | --- | --- | --- | --- | --- | --- | --- | --- | --- | --- | --- | --- | --- | --- | --- | --- | --- | --- | --- | --- | --- | --- | --- |
|  | Model 1 ^a^ | | | | | | Model 2 ^b^ | | | | | | Model 3 ^c^ | | | | | |  | Model 1 ^a^ | | | | | | Model 2 ^b^ | | | | | | Model 3 ^c^ | | | | |
|  | P value | ORs ^d^ | 95 % CI | | |  | P value | ORs ^d^ | 95 % CI | | |  | P value | ORs ^d^ | 95 % CI | | |  |  | P value | ORs ^d^ | 95 % CI | | |  | P value | ORs ^d^ | 95 % CI | | |  | P value | ORs ^d^ | 95 % CI | | |
| Overall | 0.02 | 0.97 | 0.95 | - | 1.00 | | 0.05 | 0.97 | 0.94 | - | 1.00 | | 0.03 | 0.97 | 0.94 | - | 1.00 | |  | 0.17 | 0.98 | 0.94 | - | 1.01 | | 0.21 | 0.98 | 0.94 | - | 1.01 | | 0.17 | 0.97 | 0.93 | - | 1.01 |
| Total fruits | 0.19 | 0.95 | 0.88 | - | 1.026 | | 0.19 | 0.94 | 0.86 | - | 1.03 | | 0.22 | 0.94 | 0.85 | - | 1.04 | |  | 0.20 | 0.92 | 0.81 | - | 1.04 | | 0.27 | 0.93 | 0.82 | - | 1.06 | | 0.24 | 0.92 | 0.79 | - | 1.06 |
| Total vegetables | < 0.01 | 0.77 | 0.64 | - | 0.930 | | 0.01 | 0.76 | 0.61 | - | 0.95 | | < 0.01 | 0.72 | 0.57 | - | 0.91 | |  | 0.32 | 0.85 | 0.63 | - | 1.16 | | 0.38 | 0.87 | 0.64 | - | 1.18 | | 0.33 | 0.85 | 0.61 | - | 1.18 |
| Dark colored vegetables | 0.06 | 0.86 | 0.74 | - | 1.008 | | 0.10 | 0.86 | 0.72 | - | 1.03 | | 0.15 | 0.87 | 0.72 | - | 1.05 | |  | 0.89 | 0.98 | 0.77 | - | 1.26 | | 0.94 | 0.99 | 0.77 | - | 1.27 | | 0.92 | 1.01 | 0.77 | - | 1.33 |
| Unrefined grains | 0.06 | 0.94 | 0.88 | - | 1.002 | | 0.12 | 0.94 | 0.87 | - | 1.02 | | 0.06 | 0.92 | 0.84 | - | 1.00 | |  | 0.18 | 0.93 | 0.83 | - | 1.04 | | 0.25 | 0.94 | 0.84 | - | 1.05 | | 0.11 | 0.90 | 0.80 | - | 1.02 |
| Dairy | 0.10 | 0.91 | 0.81 | - | 1.017 | | 0.06 | 0.88 | 0.78 | - | 1.00 | | 0.04 | 0.87 | 0.77 | - | 0.99 | |  | 0.07 | 0.76 | 0.57 | - | 1.02 | | 0.06 | 0.75 | 0.56 | - | 1.01 | | 0.05 | 0.73 | 0.53 | - | 1.00 |
| Total protein foods | 0.01 | 0.76 | 0.62 | - | 0.945 | | 0.03 | 0.75 | 0.58 | - | 0.97 | | 0.02 | 0.72 | 0.54 | - | 0.96 | |  | 0.74 | 1.07 | 0.73 | - | 1.55 | | 0.68 | 1.08 | 0.74 | - | 1.57 | | 0.91 | 0.98 | 0.64 | - | 1.49 |
| Plant proteins | 0.48 | 1.05 | 0.91 | - | 1.207 | | 0.17 | 1.13 | 0.95 | - | 1.33 | | 0.28 | 1.11 | 0.92 | - | 1.32 | |  | 0.65 | 1.05 | 0.85 | - | 1.30 | | 0.57 | 1.06 | 0.86 | - | 1.32 | | 0.80 | 1.03 | 0.82 | - | 1.29 |
| Unsaturated fats | 0.40 | 0.96 | 0.86 | - | 1.06 | | 0.53 | 0.96 | 0.85 | - | 1.09 | | 0.59 | 0.97 | 0.85 | - | 1.10 | |  | 0.84 | 1.02 | 0.85 | - | 1.22 | | 0.90 | 1.01 | 0.85 | - | 1.21 | | 0.98 | 1.00 | 0.82 | - | 1.22 |
| Refined grains | 0.84 | 0.99 | 0.90 | - | 1.09 | | 0.53 | 1.04 | 0.93 | - | 1.16 | | 0.44 | 1.05 | 0.93 | - | 1.19 | |  | 0.99 | 1.00 | 0.85 | - | 1.17 | | 0.79 | 1.02 | 0.87 | - | 1.21 | | 0.86 | 1.02 | 0.85 | - | 1.21 |
| Sodium | 0.55 | 1.02 | 0.95 | - | 1.10 | | 0.39 | 1.04 | 0.95 | - | 1.13 | | 0.26 | 1.05 | 0.96 | - | 1.16 | |  | 0.36 | 0.94 | 0.84 | - | 1.07 | | 0.36 | 0.94 | 0.84 | - | 1.07 | | 0.60 | 0.97 | 0.85 | - | 1.10 |
| Snack and sweets | 0.68 | 0.98 | 0.90 | - | 1.07 | | 0.52 | 0.97 | 0.87 | - | 1.07 | | 0.20 | 0.93 | 0.83 | - | 1.04 | |  | 0.97 | 1.00 | 0.86 | - | 1.15 | | 0.93 | 0.99 | 0.86 | - | 1.15 | | 0.97 | 1.00 | 0.85 | - | 1.17 |
| Saturated fats | 0.80 | 1.02 | 0.90 | - | 1.15 | | 0.58 | 0.96 | 0.83 | - | 1.11 | | 0.59 | 0.96 | 0.82 | - | 1.12 | |  | 0.47 | 1.10 | 0.85 | - | 1.41 | | 0.51 | 1.09 | 0.85 | - | 1.40 | | 0.50 | 1.10 | 0.83 | - | 1.47 |

|  | **Age < 75** | | | | | | | | | | | | | | | | | |  | **Age ≥ 75** | | | | | | | | | | | | | | | | |
| --- | --- | --- | --- | --- | --- | --- | --- | --- | --- | --- | --- | --- | --- | --- | --- | --- | --- | --- | --- | --- | --- | --- | --- | --- | --- | --- | --- | --- | --- | --- | --- | --- | --- | --- | --- | --- |
|  | Model 1 ^a^ | | | | | | Model 2 ^b^ | | | | | | Model 3 ^c^ | | | | | |  | Model 1 ^a^ | | | | | | Model 2 ^b^ | | | | | | Model 3 ^c^ | | | | |
|  | P value | ORs ^d^ | 95 % CI | | |  | P value | ORs ^d^ | 95 % CI | | |  | P value | ORs ^d^ | 95 % CI | | |  |  | P value | ORs ^d^ | 95 % CI | | |  | P value | ORs ^d^ | 95 % CI | | |  | P value | ORs ^d^ | 95 % CI | | |
| Overall | 0.29 | 0.98 | 0.95 | - | 1.01 | | 0.32 | 0.98 | 0.95 | - | 1.02 | | 0.65 | 0.99 | 0.96 | - | 1.03 | |  | 0.04 | 0.97 | 0.94 | - | 1.00 | | 0.06 | 0.97 | 0.94 | - | 1.00 | | 0.05 | 0.97 | 0.93 | - | 1.00 |
| Total fruits | 0.28 | 0.94 | 0.84 | - | 1.05 | | 0.44 | 0.96 | 0.85 | - | 1.07 | | 0.82 | 0.99 | 0.87 | - | 1.12 | |  | 0.42 | 0.97 | 0.89 | - | 1.05 | | 0.22 | 0.94 | 0.85 | - | 1.04 | | 0.11 | 0.91 | 0.82 | - | 1.02 |
| Total vegetables | 0.40 | 0.89 | 0.67 | - | 1.17 | | 0.40 | 0.88 | 0.67 | - | 1.18 | | 0.43 | 0.88 | 0.64 | - | 1.21 | |  | < 0.01 | 0.76 | 0.62 | - | 0.93 | | 0.02 | 0.77 | 0.61 | - | 0.96 | | 0.02 | 0.74 | 0.58 | - | 0.95 |
| Dark colored vegetables | 0.39 | 1.11 | 0.88 | - | 1.39 | | 0.48 | 1.09 | 0.86 | - | 1.38 | | 0.24 | 1.18 | 0.90 | - | 1.54 | |  | < 0.01 | 0.78 | 0.65 | - | 0.93 | | 0.02 | 0.79 | 0.65 | - | 0.96 | | 0.02 | 0.76 | 0.61 | - | 0.95 |
| Unrefined grains | 0.02 | 0.88 | 0.79 | - | 0.98 | | 0.02 | 0.88 | 0.79 | - | 0.98 | | 0.02 | 0.87 | 0.78 | - | 0.98 | |  | 0.44 | 0.97 | 0.90 | - | 1.05 | | 0.81 | 0.99 | 0.91 | - | 1.08 | | 0.80 | 0.99 | 0.90 | - | 1.08 |
| Dairy | 0.25 | 0.90 | 0.76 | - | 1.07 | | 0.27 | 0.91 | 0.76 | - | 1.08 | | 0.33 | 0.92 | 0.77 | - | 1.09 | |  | 0.02 | 0.86 | 0.75 | - | 0.98 | | 0.01 | 0.83 | 0.72 | - | 0.96 | | 0.01 | 0.83 | 0.71 | - | 0.96 |
| Total protein foods | 0.60 | 0.92 | 0.67 | - | 1.26 | | 0.59 | 0.91 | 0.66 | - | 1.27 | | 0.59 | 0.90 | 0.63 | - | 1.31 | |  | 0.31 | 0.89 | 0.71 | - | 1.11 | | 0.26 | 0.87 | 0.68 | - | 1.11 | | 0.27 | 0.86 | 0.66 | - | 1.12 |
| Plant proteins | 0.23 | 1.12 | 0.93 | - | 1.36 | | 0.39 | 1.09 | 0.90 | - | 1.33 | | 0.24 | 1.14 | 0.92 | - | 1.40 | |  | 0.25 | 1.10 | 0.94 | - | 1.29 | | 0.09 | 1.17 | 0.97 | - | 1.42 | | 0.08 | 1.20 | 0.98 | - | 1.47 |
| Unsaturated fats | 0.18 | 1.15 | 0.94 | - | 1.40 | | 0.22 | 1.13 | 0.93 | - | 1.38 | | 0.11 | 1.19 | 0.96 | - | 1.48 | |  | 0.13 | 0.92 | 0.82 | - | 1.03 | | 0.19 | 0.92 | 0.81 | - | 1.04 | | 0.26 | 0.93 | 0.81 | - | 1.06 |
| Refined grains | 0.54 | 0.96 | 0.83 | - | 1.11 | | 0.72 | 0.97 | 0.83 | - | 1.13 | | 0.80 | 0.98 | 0.84 | - | 1.15 | |  | 0.07 | 1.12 | 0.99 | - | 1.27 | | 0.15 | 1.11 | 0.96 | - | 1.27 | | 0.08 | 1.14 | 0.98 | - | 1.33 |
| Sodium | 0.32 | 0.94 | 0.84 | - | 1.06 | | 0.30 | 0.94 | 0.84 | - | 1.06 | | 0.62 | 0.97 | 0.85 | - | 1.10 | |  | 0.37 | 1.04 | 0.96 | - | 1.12 | | 0.39 | 1.04 | 0.95 | - | 1.13 | | 0.35 | 1.05 | 0.95 | - | 1.15 |
| Snack and sweets | 0.60 | 1.04 | 0.90 | - | 1.19 | | 0.58 | 1.04 | 0.91 | - | 1.19 | | 0.54 | 1.05 | 0.90 | - | 1.21 | |  | 0.13 | 0.92 | 0.83 | - | 1.02 | | 0.18 | 0.92 | 0.82 | - | 1.04 | | 0.05 | 0.88 | 0.77 | - | 1.00 |
| Saturated fats | 0.32 | 1.12 | 0.90 | - | 1.39 | | 0.35 | 1.11 | 0.89 | - | 1.38 | | 0.43 | 1.10 | 0.87 | - | 1.39 | |  | 0.36 | 0.93 | 0.81 | - | 1.08 | | 0.34 | 0.92 | 0.78 | - | 1.09 | | 0.54 | 0.94 | 0.79 | - | 1.13 |

|  | **Body mass index < 24** | | | | | | | | | | | | | | | | | |  | **Body mass index ≥ 24** | | | | | | | | | | | | | | | | |
| --- | --- | --- | --- | --- | --- | --- | --- | --- | --- | --- | --- | --- | --- | --- | --- | --- | --- | --- | --- | --- | --- | --- | --- | --- | --- | --- | --- | --- | --- | --- | --- | --- | --- | --- | --- | --- |
|  | Model 1 ^a^ | | | | | | Model 2 ^b^ | | | | | | Model 3 ^c^ | | | | | |  | Model 1 ^a^ | | | | | | Model 2 ^b^ | | | | | | Model 3 ^c^ | | | | |
|  | P value | ORs ^d^ | 95 % CI | | |  | P value | ORs ^d^ | 95 % CI | | |  | P value | ORs ^d^ | 95 % CI | | |  |  | P value | ORs ^d^ | 95 % CI | | |  | P value | ORs ^d^ | 95 % CI | | |  | P value | ORs ^d^ | 95 % CI | | |
| Overall | < 0.01 | 0.96 | 0.93 | - | 0.98 | | 0.03 | 0.97 | 0.94 | - | 1.00 | | 0.12 | 0.98 | 0.95 | - | 1.01 | |  | 0.18 | 0.98 | 0.94 | - | 1.01 | | 0.17 | 0.97 | 0.93 | - | 1.01 | | 0.06 | 0.96 | 0.92 | - | 1.00 |
| Total fruits | 0.03 | 0.91 | 0.84 | - | 0.99 | | 0.09 | 0.92 | 0.84 | - | 1.01 | | 0.30 | 0.95 | 0.85 | - | 1.05 | |  | 0.12 | 0.91 | 0.80 | - | 1.03 | | 0.26 | 0.93 | 0.81 | - | 1.06 | | 0.10 | 0.88 | 0.75 | - | 1.02 |
| Total vegetables | < 0.01 | 0.74 | 0.60 | - | 0.90 | | 0.08 | 0.81 | 0.64 | - | 1.03 | | 0.06 | 0.79 | 0.61 | - | 1.01 | |  | 0.04 | 0.75 | 0.57 | - | 0.98 | | 0.02 | 0.71 | 0.53 | - | 0.95 | | 0.01 | 0.65 | 0.46 | - | 0.91 |
| Dark colored vegetables | 0.05 | 0.85 | 0.72 | - | 1.00 | | 0.27 | 0.90 | 0.75 | - | 1.09 | | 0.31 | 0.90 | 0.74 | - | 1.10 | |  | 0.24 | 0.87 | 0.68 | - | 1.10 | | 0.12 | 0.82 | 0.63 | - | 1.05 | | 0.27 | 0.85 | 0.64 | - | 1.13 |
| Unrefined grains | < 0.01 | 0.89 | 0.83 | - | 0.96 | | 0.15 | 0.94 | 0.87 | - | 1.02 | | 0.51 | 0.97 | 0.88 | - | 1.07 | |  | 0.35 | 0.95 | 0.86 | - | 1.06 | | 0.45 | 0.96 | 0.86 | - | 1.07 | | 0.11 | 0.91 | 0.80 | - | 1.02 |
| Dairy | < 0.01 | 0.82 | 0.71 | - | 0.95 | | < 0.01 | 0.79 | 0.67 | - | 0.93 | | < 0.01 | 0.79 | 0.67 | - | 0.94 | |  | 0.53 | 0.95 | 0.82 | - | 1.11 | | 0.40 | 0.93 | 0.80 | - | 1.09 | | 0.21 | 0.90 | 0.76 | - | 1.06 |
| Total protein foods | 0.05 | 0.81 | 0.66 | - | 1.00 | | 0.20 | 0.86 | 0.68 | - | 1.08 | | 0.32 | 0.88 | 0.68 | - | 1.13 | |  | 0.67 | 1.09 | 0.73 | - | 1.63 | | 0.69 | 1.09 | 0.71 | - | 1.68 | | 0.78 | 1.07 | 0.67 | - | 1.71 |
| Plant proteins | 0.77 | 1.02 | 0.89 | - | 1.18 | | 0.12 | 1.14 | 0.96 | - | 1.36 | | 0.08 | 1.18 | 0.98 | - | 1.42 | |  | 0.87 | 1.02 | 0.83 | - | 1.25 | | 0.80 | 1.03 | 0.83 | - | 1.28 | | 0.51 | 1.08 | 0.85 | - | 1.38 |
| Unsaturated fats | 0.35 | 0.95 | 0.85 | - | 1.06 | | 0.52 | 0.96 | 0.85 | - | 1.09 | | 0.45 | 0.95 | 0.83 | - | 1.08 | |  | 0.52 | 1.06 | 0.88 | - | 1.28 | | 0.56 | 1.06 | 0.87 | - | 1.29 | | 0.54 | 1.06 | 0.87 | - | 1.29 |
| Refined grains | 0.92 | 1.00 | 0.91 | - | 1.09 | | 0.88 | 1.01 | 0.90 | - | 1.13 | | 0.63 | 1.03 | 0.91 | - | 1.16 | |  | 0.81 | 0.98 | 0.83 | - | 1.15 | | 0.57 | 1.05 | 0.88 | - | 1.27 | | 0.61 | 1.05 | 0.86 | - | 1.29 |
| Sodium | 0.96 | 1.00 | 0.93 | - | 1.08 | | 0.89 | 0.99 | 0.91 | - | 1.09 | | 0.97 | 1.00 | 0.91 | - | 1.10 | |  | 0.98 | 1.00 | 0.89 | - | 1.12 | | 0.89 | 0.99 | 0.88 | - | 1.12 | | 0.92 | 0.99 | 0.88 | - | 1.12 |
| Snack and sweets | 0.64 | 0.98 | 0.89 | - | 1.07 | | 0.48 | 0.96 | 0.86 | - | 1.07 | | 0.36 | 0.95 | 0.84 | - | 1.07 | |  | 0.78 | 0.98 | 0.86 | - | 1.12 | | 0.73 | 0.98 | 0.85 | - | 1.12 | | 0.49 | 0.95 | 0.80 | - | 1.11 |
| Saturated fats | 0.48 | 1.05 | 0.91 | - | 1.21 | | 0.69 | 1.03 | 0.88 | - | 1.21 | | 0.71 | 1.03 | 0.87 | - | 1.22 | |  | 0.72 | 0.97 | 0.82 | - | 1.15 | | 0.52 | 0.94 | 0.78 | - | 1.13 | | 0.52 | 0.94 | 0.77 | - | 1.15 |

ORs: Odds ratios; CI: Confidence intervals

^a^ Model 1: Unadjusted

^b^ Model 2: Adjusted by Sex, Age, and Physical activity

^c^ Model 3: Model 2 + adjusted by Smoking, Alcohol, Hypertension, Diabetes, Dyslipidemia, Survey year, Occupation, Education, Marriage, and Income

^d^ Odds ratios represent the odds of sarcopenia when the scores of these components increased by one unit.

**Supplementary figure 1**

**STROBE flow diagram: Flowchart of eligible participants for the study**

Aged 65 or older (n=1440)

Participants in 2014-2016 NAHSIT (n=8423)

Completed measurement of 8-meter walk speed

and dual energy X-ray absorptiometry (n=566)

Final analysis (n=411)

Excluded (n=155)

| ◆ | Missing information on assessment of Healthy Eating Index for Taiwanese (n=124) |
| --- | --- |
|  |  |
| ◆ | Inadequate/excessive energy intake (n=12) |
| ◆ | Prior malignant cancer diagnosis (n=19) |
